# Effectiveness of Text Messages and Text Messages Plus Peer Support on Psychiatric Readmission and Length of Stay: Outcomes From a Quantitative Stepped-Wedge Cluster Randomized Trial

**DOI:** 10.2196/81760

**Published:** 2025-11-18

**Authors:** Vincent Israel Opoku Agyapong, Reham Shalaby, Belinda Agyapong, Wanying Mao, Ernest Owusu, Hossam Eldin Elgendy, Ejemai Eboreime, Peter H Silverstone, Pierre Chue, Xin-Min Li, Wesley Vuong, Arto Ohinmaa, Frank MacMaster, Andrew J Greenshaw

**Affiliations:** 1 Department of Psychiatry Faculty of Medicine Dalhousie University Halifax, NS Canada; 2 Department of Psychiatry Faculty of Medicine and Dentistry University of Alberta Edmonton, AB Canada; 3 Addiction and Mental Health Services Alberta Health Services Edmonton, AB Canada; 4 School of Public Health University of Alberta Edmonton, AB Canada

**Keywords:** text messaging, peer support, re-admission, length of stay, in-patient, psychiatric

## Abstract

**Background:**

Mental health recovery typically continues after patients leave the hospital. However, hospital readmission in the 12 months after discharge is common and costly.

**Objective:**

This study aimed to examine the effectiveness of supportive text messaging (hereinafter “SMS”) and SMS with or without peer support service on hospital readmission and length of stay after discharge from inpatient psychiatric care.

**Methods:**

A stepped-wedge cluster randomized trial was used to examine differences in the changes in the mean number of admissions and the mean duration of total length of stay in days, for patients discharged from psychiatric inpatient care, at 6 and 12 months pre- and post index admissions, for 2 intervention periods compared to a control period of treatment as usual.

**Results:**

Overall, 1070 participants were assigned to 1 of 3 study arms: SMS (n=302), SMS with or without peer support service (n=342), or treatment as usual (n=426). Compared to treatment as usual, SMS with or without peer support service reduced hospital readmissions 6 months pre- and post index admission by an average of 0.26 admissions, and SMS alone reduced inpatient length of stays 6 months pre- and post index admission by an average of 7.28 days.

**Conclusions:**

Our results demonstrate that simple, low-cost digital tools—either by themselves or paired with peer support—can help close gaps in postdischarge care. We anticipate that these findings may inform future service delivery models and policy development aimed at enhancing postdischarge mental health support. By supporting smoother transitions and reducing future hospital use, such approaches may offer a scalable way to build more sustainable and person-centered mental health systems.

**Trial Registration:**

ClinicalTrials.gov NCT05133726; https://clinicaltrials.gov/study/NCT05133726

## Introduction

Mental health crisis often leads individuals discharged from acute psychiatric hospitals to seek readmissions, contributing to prolonged hospital stays [1-3]. Avoidable readmissions and increased length of hospital stay have emerged as a significant concern for health systems globally [4,5]. Such readmissions not only impose substantial physical, psychological, and financial burdens on patients and their families but also strain health system resources [6-9]. One study, which also examined patients in Alberta, Canada, reported that 14.0% of psychiatric hospitalizations among young adults were followed by readmission within 90 days, and the median time to readmission was 24 days [10]. High readmission rates and increased length of hospital stay consume limited infrastructural, human, and financial capacities, thereby compromising the efficiency and sustainability of health care delivery [6,11,12]. This challenge has become even more pronounced in the post–COVID-19 era, and many hospitals continue to face surges in psychiatric emergency department presentations, readmissions, and capacity pressures [13]. As a result, there is growing global interest in identifying and implementing effective strategies to reduce preventable readmissions, particularly within acute care settings [14]. Traditional interventions may not fully address the complex needs of individuals recently discharged from acute psychiatric hospitals who experience postdischarge mental health crises [1,2,15,16]. These individuals are at heightened risk for readmission, often due to inadequate postdischarge support, as traditional follow-up care models may not provide sufficient engagement or timely intervention, leading to increased reliance on emergency and acute mental health services [15]. Recent innovations, such as text messaging support and peer support programs, have emerged as promising adjuncts to conventional care, aiming to prevent psychiatric readmissions and shorten hospital stays [17,18]. These interventions offer scalable, cost-effective, and accessible solutions to enhance recovery and reduce the burden on acute psychiatric services. For example, Stevens et al [19] demonstrated that an SMS brief contact intervention significantly reduced repeat hospital presentations for self-harm among patients receiving hospital-based care. This finding supports the growing potential of SMS-based interventions to potentially improve outcomes and reduce readmission rates across diverse mental health populations [19]. Text messaging interventions offer a convenient and immediate means of communication, delivering supportive messages, reminders, and resources to individuals in real-time [20,21]. Text messaging interventions have also demonstrated significant efficacy in improving medication adherence and clinical engagement among individuals with serious mental illness [22,23]. A systematic review encompassing various psychiatric disorders reported that text messaging interventions significantly improved medication adherence and clinical outcomes in a majority of studies. These interventions were well-received by participants, highlighting their feasibility and acceptability as a component of mental health care [22].

In recent years, peer support services (PSS) have gained considerable attention as an innovative and effective complement to traditional mental health interventions [24]. Peer support involves individuals with lived experience providing emotional and practical assistance to others facing similar challenges, fostering a sense of hope and empowerment [24-26]. A systematic review and meta-analysis of one-to-one peer support interventions in mental health services, which included 23 studies reporting 19 trials and 3329 participants, further corroborated these findings [27]. The review suggested that one-to-one peer support may have a modest positive impact on self-reported recovery (relative risk=0.22, 95% CI 0.01- 0.42, *z* score=2.0, *P*=.04) and empowerment (relative risk=0.23, 95% CI 0.04- 0.42, *z* score=2.3, *P*=.02), but no impact on clinical symptoms or service use, suggesting that peer support may be more effective in enhancing personal and social functioning rather than directly altering clinical parameters [27]. It is therefore likely that the combination of peer support and text messaging may offer synergistic benefits [17,18]. Peer support provides relational and emotional benefits [24], while text messaging offers structured, timely, and scalable support [21,28]. Together, these interventions can address both the emotional and practical needs of individuals with mental health concerns, potentially leading to reduced readmission rates and decreased length of hospital stays [1,20]. Moreover, they align with recovery-oriented care models that emphasize patient-centered, community-based, and continuous support [17]. A controlled observational pilot study conducted in Edmonton, Alberta, evaluated the effectiveness of peer support and supportive text messaging on recovery outcomes among patients discharged from acute psychiatric care [17]. The study found that participants receiving the combined intervention (peer support plus text messaging) exhibited significantly higher recovery scores compared to those receiving standard care or text messaging alone. Notably, improvements were observed in areas such as personal confidence, willingness to seek help, and overall recovery, with sustained benefits up to 6 months post discharge [17]. Despite promising evidence, these interventions have several implementation challenges, such as variability in program design, lack of standardization, and limited integration into existing health care systems, impacting widespread adoption [24,28]. Furthermore, the sustainability of these programs requires adequate funding, training, and ongoing evaluation to improve program delivery and effectiveness. Addressing these barriers is crucial for realizing the full potential of peer support and text messaging in transforming mental health care delivery [17,22,29].

As health care systems continue to evolve, embracing innovative, patient-centered approaches will be essential in meeting the complex needs of individuals with mental health disorders. This study aims to examine the effectiveness of text messaging and peer support on patients discharged from inpatient psychiatric hospitals by assessing the mean number of readmissions and length of stay at 6- and 12-months in a cluster randomized controlled trial. The specific objectives of the study are to determine if there are statistically significant differences in the mean changes in the number of readmissions and the mean changes in the total length of stay for patients 6 and 12 months from pre- to post index admission in three arms of the study: text messaging arm (hereinafter “SMS”), SMS with or without peer support arm, and treatment as usual arm. This study seeks to inform clinical practice and policy development and contribute to advancing mental health care strategies that are both effective and accessible.

## Methods

### Study Design and Data Collection

**Figure 1:**
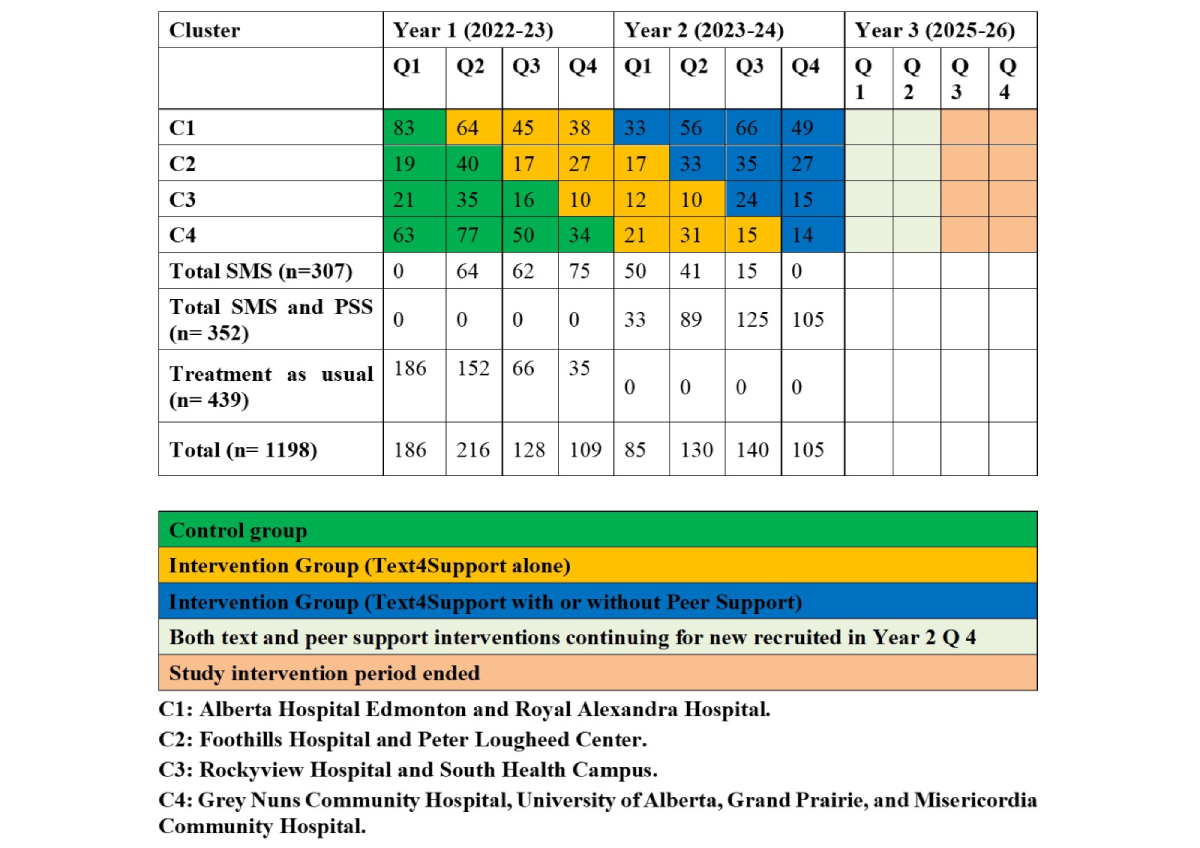
Study recruitment cluster and intervention group allocation. PSS: peer support service; SMS: supportive text messaging.

This study evaluated the effectiveness of two interventions that are (1) web-based, fully automated supportive text messages—Text4Support (SMS) alone—and (2) SMS with or without the addition of PSS, on the changes in the mean number of readmissions and length of stay among patients with psychiatric illness in the province of Alberta. A pragmatic stepped-wedge cluster-randomized approach with three arms was applied, providing usual postdischarge care alone, SMS plus usual care, and SMS with or without PSS plus usual care to the participants recruited across 10 acute care sites across Alberta as the clustered unit of randomization. This design was found to be successful with complex programs and change management involving large-scale programs [30]. The 3-arm design in this study allows for an assessment of not only whether SMS improves outcomes compared with usual care, but also whether the addition of PSS provides any incremental advantage. If combined support offers no additional benefit over SMS alone, then the SMS-only program would be the more economical and scalable option for patients discharged from acute mental health care. Conversely, if combined support clearly outperforms both SMS alone and usual care, then health authorities may consider implementing the more resource-intensive dual intervention. Randomization of the 4 cluster units was undertaken by an independent statistician. The study design and recruitment procedures have been fully described in the published study protocol [31]. No blindness was applied, and study participants were aware of their study cluster and randomization. In addition, no changes to the trial design or interventions were made after participant recruitment commenced. All patients have received concomitantly the usual care in the community (outside the scope of the study). For this study, sociodemographic characteristics were collected, including chronological age and gender; clinical data collected included primary diagnosis during hospitalization. Health usage data were obtained from Alberta Health Services administrative data sources spanning 1 year before the index admission date and 1 year post discharge. Individual deidentified participant data was applied by removing patients’ names, and the primary identifier was the patient’s phone number and health identifying number. Figure 1 depicts the unit cluster randomization.

### Ethical Considerations

The ethical requirement for this study was provided by the University of Alberta’s Health Research Ethics Board (Ref number Pro00111459). In addition to ethics, the regional health authority also provided additional operational approval. All participants involved in this project provided written informed consent, and the privacy and confidentiality of participants were guaranteed by not including any participant information in published manuscripts and reporting data as aggregates. The research was conducted in accordance with the Declaration of Helsinki. No financial compensation was provided to the participants.

### Participant Recruitment

Study participants were recruited among individuals who had been diagnosed with a mental illness and were ready for discharge from the inpatient psychiatry units. Face-to-face discussions were conducted to recruit participants across 10 major acute psychiatric care sites in Edmonton, Calgary, and Grand Prairie in Alberta. Operational managers and clinical staff supported the research team by identifying patients about to be discharged within 7 days from a psychiatric unit. Eligible patients were provided with detailed study information. Written consent was obtained, and participants were invited to complete a self-administered questionnaire on a tablet device. The inclusion criteria included being above 18 years old, having had a mobile device, being able to read English text messages, and being able to provide consent to participate in the study. Exclusion criteria involved the participants who knew they would be based out of town during the 12-month follow-up period. The recruitment period was extended for 2 years, starting in February 2022 and ending in February 2024. Study participants were allocated into one of 3 intervention groups based on cluster randomization. There were 1155 study participants recruited across the entire recruitment period. Psychiatrists and nursing staff helped identify patients likely to be readmitted post discharge and eligible for PSS. This approach aligns with the principles of pragmatic trials, where clinical judgment and team-based risk evaluation inform the allocation of postdischarge PSS [32].

### Text and Peer Support Interventions

The Text4Support program delivered through the ResilienceNHope platform [33] offered evidence-based daily supportive text messages to individuals discharged from acute psychiatric care. This low-cost program, like it is predecessor Text4Hope program, which enrolled over 50,000 subscribers during the COVID-19 pandemic [34], aimed to reduce the psychological treatment gap and was used alone or in combination with peer support. The evaluation of the Text4Hope program showed that automated, web-based daily supportive text messages are effective in reducing psychological distress among the general public and received high levels of user satisfaction [35-37]. Messages began the day after enrollment and were sent once daily in a unidirectional (no-reply) format. These messages were crafted based on cognitive behavioral therapy principles by mental health clinicians in collaboration with individuals who have lived experience. The messages provided general support (hope, affirmation, and self-care) as well as diagnosis-specific content based on 6 common psychiatric diagnoses: mood disorders, anxiety disorders, psychotic disorders, substance use disorders, adjustment disorders, and personality disorders. For instance, some messages for depression focused on behavioral activation, while those for anxiety emphasized relaxation techniques. The first text message participants received included the phone number for mental health crisis services they could contact if needed. Participants assigned to the Text4Support program had their phone numbers registered in the ResilienceNHope application to receive 6 months of free, daily, web-based automated supportive text messages tailored to their primary mental health concern. The peer support intervention was delivered by PSSs—individuals with lived experience of mental illness who are in recovery themselves. Their responsibilities included face-to-face visits, virtual interactions (via phone, text, or Zoom), advocacy, linking patients with community resources, and sharing personal recovery experiences.

### Sample Size Considerations

We estimated that the sample size needed to assess the effects of the SMS and PSS interventions on the outcome variables would be 1051. This was calculated with a projection that the effect size for the reduction in mean scores of readmissions and length of stay (LOS) at 6 months from baseline would be 0.2, a population variance of 1 for each mean score, and a 2-sided significance level α=.05, and a power of 90% (β=.1).

### Outcome Measures

The study's primary outcomes involved health usage parameters, including differences in the mean changes in the number of readmissions and LOS 6 and 12 months before admission preadmission and post discharge among the study intervention. Outcome measures were extracted from Alberta Health Services administrative data sources.

### Statistical Analysis

Data analysis was performed using SPSS (IBM Corp) for Windows, Version 25 [38]. Participants’ study intervention groups were plotted against all independent variables. Chi-square or Fisher exact test was used for categorical variables; one-way ANOVA was conducted to determine if there were significant differences in the mean age of participants in the 3 treatment groups. A difference-in-difference analysis was conducted to examine the effect of the interventions on the mean difference in the outcome variables from preadmission to follow-up respective time points (eg, LOS 6 and 12 months pre-enrollment to 6 and 12 months post discharge, respectively). Considering violations of ANOVA assumptions—specifically, nonnormality of data and heterogeneity of variances—we applied the Welch *F* test. Effect sizes were estimated using an omega-squared statistic adjusted for the Welch degrees of freedom (ω² = [df₁ (F − 1)] / [df₁ (F − 1) + N]). Post hoc comparisons were conducted using the Games–Howell test. The statistical analysis plan for this study, which is based on means and SDs and incorporates the preindex admission event, is grounded in the theoretical and conceptual framework of adverse events in psychiatry—such as self-harm [39], emergency department visits [40], and inpatient admissions [41-44]—where a previous history of the event is among the strongest predictors of recurrence. For example, it has been reported that patients with previous psychiatric hospitalizations before an index admission have twice the risk of readmission within 12 months compared to those without such a history [43].

Percentages and raw data were used to report the descriptive characteristics, with a significance level of *P*.05 used to determine statistical significance for all analyses. Regarding the missing data, there were a total of 28 missed cases (SMS: n=5; SMS with or without PSS: n=10, and TAU: n=13). The administrative data related to the index admission of these patients were not available at the health authority (Alberta Health Services). Data were missed from each cluster at random; possible reasons were not a valid health care number, an out-of-province health care number, an invalid phone number, or an inability to locate the index admission in health records. In addition, 6 patients dropped out of the study (equally distributed among the clusters). No imputation of missing data was applied, and the analysis was run with the intention to treat, that is, the participants were deemed adherent or receiving the intended intervention regardless of the actual receipt of the service.

## Results

**Figure 2:**
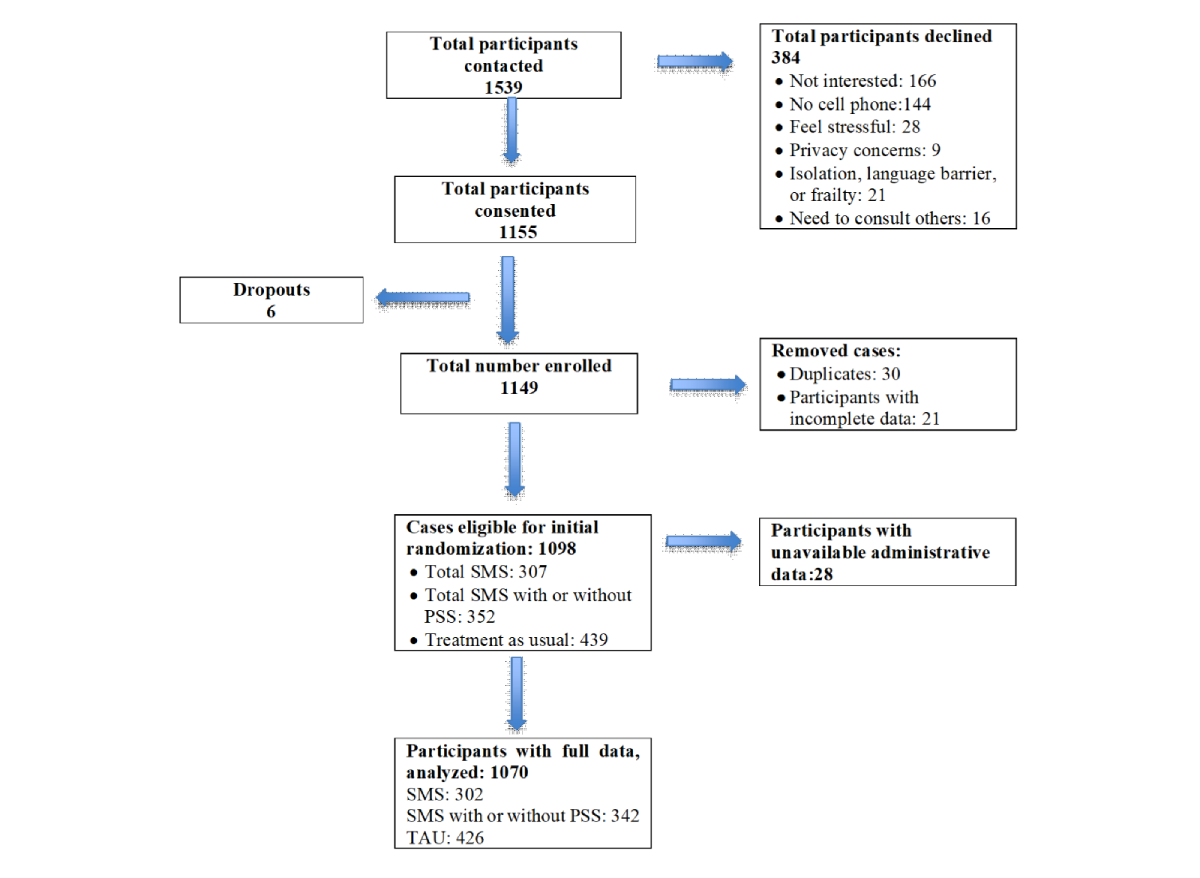
Study flow diagram. PSS: peer support service; SMS: supportive text messaging; TAU: treatment as usual.

Figure 2 shows the study flowchart.

Overall, 1070 participants were assigned to one of 3 study arms: supportive text messaging alone (SMS, n=302), supportive text messaging with or without peer support (SMS with or without PSS, n=342), or treatment as usual (TAU, n=426). Table 1 illustrates the results related to participants’ demographic characteristics and clinical data in relation to the study intervention groups. Out of 1070 participants, 395 (36.9%) of participants were younger than 25 years, 586 (54.8%) were females, and had a primary diagnosis of depression or anxiety (639/1070, 59.8%). The rest had a primary diagnosis of substance use disorder, personality disorder, or other conditions (257/1070, 24.0%), and psychosis (173/1070, 16.2%). There was a significant difference among the intervention groups regarding the age of the participants when assessed both as a continuous variable (*F*_2,10467_=18.53, *P*<.01) and as a categorical variable (*χ*^2^_4_41.64, *P*<.01). There were no significant differences between the intervention groups with respect to gender and primary diagnostic category.

Table 2 demonstrates changes in the mean number of admissions and LOS 6 and 12 months pre- and 6 and 12 months post index admission. The table showed that there was an overall reduction of the health usage parameters post discharge except for the LOS among the SMS with or without PSS and TAU groups, regardless of the follow-up time point.

Table 3 illustrates the effectiveness of study interventions on changes in health usage parameters 6 and 12 months pre- and post discharge. The results were evaluated using the Welch *F* test, and effect sizes were estimated using an omega-square statistic adjusted for the Welch degrees of freedom (ω² = [df₁ (F − 1)] / [df₁ (F − 1) + N]). From the table, there was a significant difference in changes in health usage among different study groups as reported for the mean difference in the number of admissions 6 months pre- and post index admission (*F*_2,1032_= 4.05, *P*=.02), with a small effect size (ω²<.01) and the change in mean LOS 6 months pre- and post index admission (*F*_2,1032_=3.95, *P*=.02, with a small effect size (ω²<.01).

Table 4 depicts the post hoc analysis (pairwise comparison) among the study intervention groups regarding the mean changes in the number of admissions between the study groups 6 months pre and post index admission. From the table, there was a significantly greater reduction in mean change in number of admissions for the SMS with or without PSS group when compared to the TAU group (mean difference 0.26, 95% CI value 0.05-0.48). Whilst the mean reduction in the number of admissions for the SMS group was much larger than for the TAU group, the difference in the changes in mean number of admissions between the two groups did not reach statistical significance (*P*>.05). Similarly, there was no statistically significant differences between the SMS group and the SMS with or without PSS group with respect to the mean changes in the number of admissions pre and post the index admission.

Table 5 depicts the post hoc analysis (pairwise comparison) among the study groups regarding the difference in values of the total LOS from 6 months preadmission to 6 months after discharge. There was a significant reduction in the mean LOS pre and post index admission for the SMS group as compared to the TAU group, with (mean difference of 7.28 (95% CI value 1.08-13.48). However, there were no statistically significant differences between the SMS group and the SMS with or without PSS group, or the SMS with or without PSS group and the TAU group with respect to the mean changes in the LOS pre and post the index admission.

**Table 1:** Distribution of age, gender, and diagnostic category among the study group participants.

Variables, n (%)			Intervention groups				Value (*df*)	*P* value
			SMS^a^ (N=302)	SMS with or without PSS^b^ (N=342)	TAU^c^ (N=426)	Total (N=1070)		
**Chi-square test**							41.64 (4)	<.01
	**Age (years), n (%)**							
		≤25	111 (36.8)	169 (49.4)	115 (27.0)	395 (36.9)		
		26-40	105 (34.8)	97 (28.4)	163 (38.3)	365 (34.1)		
		>40	86 (28.5)	76 (22.2)	148 (34.7)	310 (29.0)		
	**Gender, n (%)**						2.96 (4)	.57
		Male	121 (40.1)	140 (40.9)	191 (44.8)	452 (42.2)		
		Female	174 (57.6)	190 (55.6)	222 (52.1)	586 (54.8)		
		Other gender	7 (2.3)	12 (3.5)	13 (3.1)	32 (3.0)		
	**Primary mental health diagnosis, n (%)**						0.44 (4)	.98
		Depression or anxiety	180 (59.8)	204 (59.6)	255 (59.9)	639 (59.8)		
		Psychosis	46 (15.3)	58 (17.0)	69 (16.2)	173 (16.2)		
		Substance use disorder, personality disorder or other	75 (24.9)	80 (23.4)	102 (23.9)	257 (24.0)		
**ANOVA Test, (F test)**								
	Age (years), mean (95% CI values)		33.57 (32.04-35.10)	30.90 (29.52-32.29)	36.97 (35.57-38.36)	34.07 (33.23-34.91)	18.53 (2,1067)	<.01

^a^SMS: supportive text messaging.

^b^PSS: peer support service.

^c^TAU: treatment as usual.

**Table 2:** Changes in mean number of admissions and LOS^a^ 6 and 12 months pre- and 6 or 12 months post index admission.

Intervention arm		6 months, mean (SD)			12 months, mean (SD)		
		Pre	Post	Mean change (post-pre)	Pre	Post	Mean change (post-pre)
**Changes in the mean number of admissions 6 and 12 months pre- and 6 and 12 months post index admission**							
	SMS^b^	0.90 (1.08)	0.45 (1.02)	–0.42 (1.31)	1.26 (1.48)	0.79 (1.47)	–0.44 (1.72)
	SMS with or without PSS^c^	1.03 (1.12)	0.44 (1.04)	–0.56 (1.23)	1.29 (1.53)	0.74 (1.48)	–0.58 (1.77)
	TAU^d^	0.82 (1.05)	0.52 (1.13)	–0.30 (1.37)	1.18 (1.61)	0.76 (1.48)	–0.41 (1.70)
**Changes in mean inpatient LOS 6 and 12 months pre- and 6 and 12 months post index admission**							
	SMS	7.98 (21.10)	7.21 (24.71)	–0.54 (29.71)	13.49 (29.50)	12.48 (29.96)	–0.70 (36.75)
	SMS with or without PSS	6.48 (17.40)	10.80 (36.73)	3.69 (34.77)	10.95 (26.02)	15.54 (41.16)	3.35 (39.56)
	TAU	6.57 (15.56)	13.27 (41.26)	6.74 (42.19)	12.40 (26.20)	17.48 (44.13)	5.55 (47.24)

^a^LOS: length of stay.

^b^SMS: supportive text messaging.

^c^PSS: peer support service.

^d^TAU: treatment as usual.

**Table 3:** Welch F test results exploring the difference-in-difference of the mean changes in the health usage parameters among study participants 6 and 12 months pre- and post index admission.

Outcome variable (Welch *F* Test)	SMS^a^ (6 and 12 months), mean difference (95% CI values)	SMS with or without PSS^b^, 6 and 12 months, mean difference (95% CI values)	TAU^c^, 6 and 12 months, mean difference^d^ (95% CI values)	*F* value (*df*)	*P* value	Omega square (ω²)
6 months admission	–0.42 (–0.57 to –0.27)	–0.56 (–0.69 to –0.43)	–0.30 (–0.43 to –0.17)	4.05 (2)	.02	<.01
12 months admission	–0.44 (–0.64 to –0.25)	–0.58 (–0.77 to –0.40)	–0.41 (–0.57 to –0.25)	1.07 (2)	.34	<.01
6 months LOS	–0.54 (–3.86 to 2.79)	3.69 (0.84 to 7.29)	6.74 (2.75 to 10.73)	3.95 (2)	.02	<.01
12 months LOS^e^	–0.70 (–4.82 to 3.41)	3.35 (–0.75 to 7.44)	5.55 (1.09 to 10.02)	2.16 (2)	.12	<.01

^a^SMS: supportive text messaging.

^b^PSS: peer support service.

^c^TAU: treatment as usual.

^d^Mean difference: Indicates the mean of the net difference from pre to post index admission time points (eg, 6 months or 12 months).

^e^LOS: length of stay.

**Table 4:** Pairwise comparison of the mean difference in the number of admissions 6 months pre- and post index admission among the study groups using Games-Howell post hoc test.

(I)^a^ and (J)^b^ intervention types		Mean difference (I–J)	SE	*P* value	95% CI for difference
**SMS^c^**					
	SMS with or without PSS	0.140	0.099	.33	–0.091 to 0.372
	TAU^d^	–0.122	0.099	.44	–0.355 to 0.111
**SMS with or without PSS^e^**					
	SMS	–0.140	0.099	.33	–0.372 to 0.091
	TAU	–0.263^f^	0.092	.01	–0.479 to –0.046
**TAU**					
	SMS	0.122	0.099	.44	–0.111 to 0.355
	SMS with or without PSS	0.263^f^	0.092	.01	0.046 to 0.479

^a^Intervention type selected for comparison with two other intervention types.

^b^Intervention types being compared against the selected intervention.

^c^SMS: supportive text messaging.

^d^TAU: treatment as usual.

^e^PSS: peer support service.

^f^Significant findings

**Table 5:** Pairwise comparison of the mean difference in the LOS^a^ 6 months pre- and post index admission among the study groups using the Games-Howell post-hoc test.

(I)^b^ and (J)^c^ intervention types		Mean difference (I–J)	SE	*P* value	95% CI for difference
**SMS^d^**					
	SMS with or without PSS^e^	–4.225	2.493	.21	–10.080 to 1.631
	TAU^f^	–7.279^g^	2.642	.02	–13.482 to –1.076
**SMS with or without PSS**					
	SMS	4.225	2.493	.21	–1.631 to 10.080
	TAU	–3.054	2.735	.50	–9.476 to 3.367
**TAU**					
	SMS	7.279^g^	2.642	.02	1.076 to 13.482
	SMS with or without PSS	3.054	2.735	.50	–3.367 to 9.476

^a^LOS: length of stay.

^b^Intervention type selected for comparison with two other intervention types.

^c^Intervention types being compared against the selected intervention.

^d^SMS: supportive text messaging.

^e^PSS: peer support service.

^f^TAU: treatment as usual.

^g^Significant findings.

## Discussion

### Principal Findings

This cluster randomized controlled trial evaluated the effectiveness of supportive text messaging and peer support on psychiatric readmissions and hospital LOS at 6 and 12 months following discharge from acute inpatient psychiatric care. Applying the Welsh F test, there was a statistically significant reduction in mean readmissions was observed in the SMS with or without PSS group compared to the TAU group (*F*_2,1032_= 4.05, *P*=.02), with a mean difference of 0.26 admissions (mean difference=0.263; 95% CI 0.046-0.479). Additionally, the SMS group demonstrated a significant reduction in LOS at 6 months post discharge compared to the TAU group (*F*_2,1032_=3.95, *P*=.02), with a mean difference of 7.28 days (95% CI 1.076-13.482) and a small effect size (ω²<.01). The differences in changes in mean admissions and LOS between the 3 groups 12 months pre- and post index admission were not statistically significant. These findings suggest that supportive text messaging, with or without peer support, may be effective in reducing both psychiatric readmissions and hospitalization duration during the critical early period following discharge. The result is consistent with previous studies demonstrating that SMS interventions can enhance continuity of care, medication adherence, and engagement with services, all of which may contribute to earlier symptom recognition and intervention, thus potentially shortening hospital stays [23,45]. Despite the modest effect sizes, the low cost, scalability, and ease of integration of SMS-based interventions make them an attractive adjunct to traditional mental health services. Notably, the SMS with or without PSS group showed significant differences in the mean change in readmission numbers at 6 months but not at 12 months, and no change in the LOS at either 6 or 12 months compared to the TAU group. This may reflect implementation variability, insufficient peer support exposure, or differences in participant engagement. Although peer support may positively affect psychosocial outcomes such as empowerment and perceived recovery [25], its effect on clinical service usage outcomes (eg, LOS, readmissions) has been inconsistent. For instance, a study assessing peer support interventions aimed at reducing psychiatric readmissions within one year of discharge found that providing one-to-one peer support in addition to usual care for patients at risk of readmission did not demonstrate superiority over usual care alone during the 12-month follow-up period [32]. Variability in peer training, frequency of contact, and integration into clinical teams are potential contributing factors that warrant further investigation [24-26].

The absence of statistically significant differences in the mean change in readmissions at both 6 and 12 months between the SMS and TAU groups suggests that SMS intervention alone may not sufficiently address the complex, multifactorial causes of psychiatric rehospitalization. Factors such as housing instability, substance use, social support deficits, and fragmented community services play a substantial role in driving readmissions [1-3,16]. While text messaging and peer support may improve engagement and subjective recovery, their ability to reduce reliance on acute care likely requires integration with more comprehensive, individualized, and coordinated post-discharge services [1,16]. Despite these limitations, the significant reduction in LOS at six months for the SMS group and the significant reduction in the readmission rate at 6 months for the SMS with or without PSS group compared to the TAU group indicate that even modest digital interventions and peer support can positively affect clinical outcomes. This aligns with recovery-oriented models of care that prioritize accessible, continuous, and patient-centered support following hospital discharge [2,17,46]. Moreover, these findings underscore the potential for technology-based and peer-driven interventions to complement existing care structures, particularly in resource-constrained environments.

### Limitations

Several limitations should be acknowledged. First, there is a potential for selection bias, as patients offered peer support were chosen by the inpatient team based on their perceived higher risk of readmission. Second, the lack of fidelity assessments—particularly for the peer support component—limits the ability to draw firm conclusions about the effectiveness of this intervention; further research is needed to better evaluate its potential effectiveness. Third, the study did not assess or control for outpatient treatments received by participants, which can be a significant potential confounding factor influencing readmission rates. Fourth, the achieved sample size of 1070 was substantially lower than the 10,000 participants projected in the published study protocol, which, at face value, may raise concerns regarding the generalizability of the findings. However, the sample size exceeded the estimated requirement of 1051 participants needed to detect mean differences in outcome variables with 90% power at a 2-sided significance level (α) of .05, which supports the generalizability of our study findings. Fifth, while the study protocol inadvertently listed 9 study sites, the trial registration accurately identified 11. In practice, ten sites ultimately participated, as one site (Fort McMurray) was unable to proceed due to insufficient personnel to support the research activities. Despite these limitations, the findings suggest that text messaging and PSS may offer promising avenues for enhancing follow-up care for individuals discharged from inpatient psychiatric facilities. Whilst mean readmission and mean LOS after an index admission have been previously studied in the psychiatric literature [47], this study adds to the literature by examining changes in the mean admissions and LOS pre- and post index admission.

### Policy Implications and Future Directions

The results of this study carry implications for mental health policy and service planning. The observed reduction in hospital LOS at six months among participants receiving SMS compared to the TAU group underscores the potential of low-cost, scalable digital interventions to optimize post-discharge care. Similarly, the statistically significant reduction in mean readmissions observed at six months in the SMS with or without PSS group compared to the TAU group suggests there may be potential benefits for incorporating SMS and PSS into postdischarge care. However, the small effect sizes suggest that the clinical effectiveness, although present, may be limited without broader systemic changes. Notwithstanding, in resource-limited settings, where access to intensive follow-up services may be constrained, integrating SMS-based support and PSS into routine discharge planning may offer a cost-effective strategy to improve continuity of care and reduce inpatient service usage.

Health system planners and policymakers should consider formally incorporating SMS and PSS into standard discharge protocols for psychiatric inpatients. Given the minimal infrastructure required and the high penetration of mobile phone usage, particularly in underserved populations [21,28], SMS interventions represent a feasible adjunct to existing services. Their integration could also contribute to broader health equity goals by improving engagement and outcomes among individuals who face barriers to traditional in-person follow-up care. However, the lack of observed significant differences in changes in readmission rates between the SMS and TAU groups highlights the need for caution in relying on stand-alone digital interventions. Policymakers should avoid viewing such interventions as substitutes for comprehensive care. Instead, such interventions may be positioned as components within a broader, multilayered system of postdischarge support that includes PSS, housing assistance, substance use services, case management, and community-based psychosocial resources. Efforts should also be made to standardize the implementation and monitoring of peer support programs. Variability in delivery may dilute potential benefits. To enable standardization, policy initiatives aimed at developing certification programs, structured peer training, and clear role definitions could enhance the consistency and effectiveness of peer-delivered services in mental health systems.

Future research should prioritize assessment of implementation fidelity of peer support interventions, with particular attention to optimal intervention intensity, training standards, supervision quality, and integration within clinical teams. Identifying the conditions under which peer support is most effective is essential, especially given the null findings in the combined SMS and peer support arm of this study. To better understand the mechanisms by which supportive text messaging contributes to reduced LOS, mixed methods research is needed. Exploring user engagement patterns, perceptions of message relevance, and overall patient experiences can guide the refinement of message content, frequency, and delivery strategies to maximize effectiveness.

Further research should investigate whether specific subgroups—such as individuals with co-occurring substance use disorders, unstable housing, or frequent hospitalizations—derive greater benefit from such interventions. Tailored approaches for higher-risk populations may improve outcomes and promote equity in postdischarge care. Given the scalability and low cost of SMS interventions, patient experience will be important for optimization and moving toward co-creation for innovative solution pathways involving text and PSS. In addition, proactive consideration of formal economic evaluations is warranted to quantify potential cost savings associated with reduced LOS and other health care usage metrics. Demonstrating cost-effectiveness is essential to inform policy decisions and support the integration of these interventions into publicly funded mental health systems. Additionally, future studies should explore the effectiveness of multicomponent interventions that combine SMS and peer support with other services, such as case management, telepsychiatry, and community outreach. These more comprehensive models may be better suited to address the multifaceted challenges contributing to psychiatric readmission. Longitudinal studies with extended follow-up and detailed process evaluations are also needed to assess the sustainability of intervention effects and elucidate long-term outcomes. Collecting in-depth qualitative data from service users will be critical for improving the acceptability, engagement, and person-centeredness of these strategies. By addressing these research priorities, future work can contribute to the development of integrated, evidence-informed care models that harness both technological and peer-based resources to support recovery, reduce hospital dependency, and strengthen the continuum of mental health care.

### Conclusions

Overall, the results suggest that supportive text messaging and peer support may potentially serve as a valuable adjunct to traditional care. However, their greatest potential likely lies in their integration within broader, individualized, and coordinated care pathways. These findings contribute to the growing evidence base for technology-assisted mental health interventions and PSS and support their inclusion in recovery-oriented models of care. While the combination of text messaging and peer support showed modest medium-term benefits in this trial, both interventions hold promise as components of a comprehensive, recovery-oriented discharge planning approach. These findings may inform future service delivery models and policy development aimed at enhancing post-discharge mental health support.

## Appendix

Multimedia Appendix 1 CONSORT-eHEALTH checklist (V 1.6.1).

## Abbreviations

LOS: length of stay
PSS: peer support service
TAU: treatment as usual

## Data Availability

The datasets generated or analyzed during this study are available from the corresponding author on reasonable request.
